# *IL10* Haplotype Associated with Tuberculin Skin Test Response but Not with Pulmonary TB

**DOI:** 10.1371/journal.pone.0005420

**Published:** 2009-05-01

**Authors:** Thorsten Thye, Edmund N. Browne, Margaret A. Chinbuah, John Gyapong, Ivy Osei, Ellis Owusu-Dabo, Norbert W. Brattig, Stefan Niemann, Sabine Rüsch-Gerdes, Rolf D. Horstmann, Christian G. Meyer

**Affiliations:** 1 Bernhard Nocht Institute for Tropical Medicine, Department of Molecular Medicine, Hamburg, Germany; 2 University Hospital Schleswig-Holstein, Campus Lübeck, Institute of Medical Biometry and Statistics, Lübeck, Germany; 3 School of Medical Sciences, Department of Community Health, Kwame Nkrumah University of Science and Technology, Kumasi, Ghana; 4 Health Research Unit, Ministry of Health, Accra, Ghana; 5 Kumasi Centre for Collaborative Research in Tropical Medicine, Kumasi, Ghana; 6 National Reference Center for Mycobacteria, Research Center Borstel, Borstel, Germany; Institut de Pharmacologie et de Biologie Structurale, France

## Abstract

Evidence from genetic association and twin studies indicates that susceptibility to tuberculosis (TB) is under genetic control. One gene implicated in susceptibility to TB is that encoding interleukin-10 (*IL10*). In a group of 2010 Ghanaian patients with pulmonary TB and 2346 healthy controls exposed to *Mycobacterium tuberculosis*, among them 129 individuals lacking a tuberculin skin test (PPD) response, we genotyped four *IL10* promoter variants at positions −2849 , −1082 , −819 , and −592 and reconstructed the haplotypes. The *IL10* low-producer haplotype −2849A/−1082A/−819C/−592C, compared to the high-producer haplotype −2849G/−1082G/−819C/−592C, occurred less frequent among PPD-negative controls than among cases (OR 2.15, CI 1.3–3.6) and PPD-positive controls (OR 2.09, CI 1.2–3.5). Lower IL-10 plasma levels in homozygous −2849A/−1082A/−819C/−592C carriers, compared to homozygous −2849G/−1082G/−819C/−592C carriers, were confirmed by a IL-10 ELISA (p = 0.016). Although we did not observe differences between the TB patients and all controls, our results provide evidence that a group of individuals exposed to *M. tuberculosis* transmission is genetically distinct from healthy PPD positives and TB cases. In these PPD-negative individuals, higher IL-10 production appears to reflect IL-10-dependent suppression of adaptive immune responses and sustained long-term specific anergy.

## Introduction

Innate and adaptive defense mechanisms contribute to anti-*MycobacteriuM. tuberculosis* immunity. The view that successful infection control mostly depends on adaptive responses reflects observations made with the intradermal application of tuberculin (purified protein derivative; PPD). Most contacts of tuberculosis (TB) patients develop a delayed-type hypersensitivity reaction to tuberculin and are, if remaining healthy, considered protected from active TB. As the skin papule indicating PPD positivity contains reactive T lymphocytes, protection is believed to result from acquired T-cell-mediated immunity. PPD negativity can result from either lack of previous exposure, anergy due to overwhelming TB, or from any form of immunosuppression. In addition, antigen-specific tuberculin anergy in patients with pulmonary TB in the course of the disease and persisting after successful treatment has been reported [1]. PPD negativity may also indicate innate immunity after exposure without induction of adaptive mechanisms. Taken together, three scenarios are conceivable after *M. tuberculosis* infection. After uptake of the pathogen by alveolar macrophages, i) the pathogen may be destroyed in a first line of defence without inducing adaptive T-cell immune responses (PPD negativity), or ii) infection establishes and adaptive T-cell responses result in containment of bacteria (PPD positivity), or iii) innate and adaptive immune responses fail, allowing for outgrowth and spread of bacteria (disease). A firm molecular basis of the three scenarios remains to be defined.

A substantial influence of the human genetic constitution on TB susceptibility has been shown [2]. Early reports of familial hereditary and clustered disease, data from accidental application of virulent *M. tuberculosis* during a BCG vaccination campaign [3], first outbreaks in indigenous populations without prior experience of TB and outbreaks in closed environments [4]–[6], higher concordance of TB in monozygotic than in dizygotic twins [7]–[9], and family linkage and case-control association studies [10]–[12] underline that host genetic factors contribute to the outcome of *M. tuberculosis* infection. Furthermore, ethnic differences in TB susceptibility argue for a predisposing component of the human genetic make-up in disease susceptibility [6], [13].

One of the genes implicated in TB susceptibility in several studies is that encoding interleukin 10 (IL-10; OMIM 124092). Association studies performed so far of *IL10* variants in TB have yielded ambiguous results. Heterozygosity of the −1082 variant was associated with an increased TB risk in Cambodia [14], and the −592 C allele with a decreased TB risk in Korea [15]. In a population from Hongkong, the −1082G/−819C/−592C haplotype was weakly associated with relapses of pulmonary TB and with extrapulmonary TB [16]. No influence of *IL10* promoter variants on the occurence of TB in HIV-negative individuals was observed in Malawi and Spain [17], [18]. In a recent study from Turky, the the GCC and ACC haplotype distribution differed between TB cases and controls [19].

It is established that distinct *IL10* promoter haplotypes correlate with the transcriptional activity of *IL10*, whereby high, intermediate and low IL-10 production is associated with the *IL10* −1082/−819/−592 combinations GCC, ACC and ATA, respectively [20]–[22]. We hypothesized that *IL10* promoter variants might be involved in the quality of innate immune responses. Therefore, we assessed the frequencies of four promoter variants and their significance in HIV-negative TB patients and compared them with healthy exposed controls in a population from Ghana, West Africa. Controls were stratified according to their PPD reactivity and grouped into subgroups of PPD-positive and PPD-negative participants. This allowed to address innate immunity to TB by comparisons of cases versus controls, PPD-positive versus PPD-negative controls, and cases plus PPD-positive versus PPD-negative controls. Any influence exerted by the *IL10* variants on the extent of T-cell responses should be reflected in our sample of sputum-positive TB cases that we recruited in Ghana and compared it to a control group of significant size.

## Materials and Methods

### Ethics Statement

The study protocol was approved by the Committee on Human Research, Publications and Ethics, School of Medical Sciences, Kwame Nkrumah University of Science and Technology, Kumasi, Ghana, and the Ethics Committee of the Ghana Health Service, Accra, Ghana. Blood samples were taken only after a detailed explanation of the aims of the study, and consent was obtained by signature or thumbprint.

### Patients and controls

Participants were consecutively enrolled in Ghana, West Africa, between September 2001 and July 2004 at Korle Bu Teaching Hospital in Accra, Komfo Anokye Teaching Hospital in Kumasi, plus 15 additional hospitals and polyclinics in Accra and Kumasi and at regional district hospitals. The case group included of 2010 HIV-negative individuals with smear-/culture-positive pulmonary TB. Out of a total of 2346 control individuals, 1211 were unrelated personal household contacts of cases and 1135 were individuals from neighbouring houses or working contacts of cases. Cases and controls belonged to the ethnic groups of Akan (Ashanti, Fante, Akuapem), Ga-Adangbe, Ewe, all in the south of Ghana, and several other ethnic groups of northern Ghana. The proportions of ethnicities among patients and controls are given in Table 1 and ethnicities were included as a correction factor in all statistical analyses.

**Table 1:** Distribution of Ghanaian ethnic groups in TB cases and controls.

Ethnicity	TB cases	controls	
		PPD positive	PPD negative
	N (%)	N (%)	N (%)
Akan	1279 (63.6)	1292 (58.2)	95 (74.8)
Gaa	292 (14.5)	456 (20.6)	9 (7.1)
Northerners	259 (12.9)	229 (10.3)	16 (12.6)
Ewe	142 (7.1)	214 (9.6)	5 (3.9)
Unknown ethnicity	38 (1.9)	28 (1.3)	2 (1.6)

N, number of individuals. The group of Northeners comprises members of several ethnicities of Northern Ghana. All statistical analyses were corrected for ethnicities.

Phenotyping of patients was based on the medical histories and documentation of major symptoms on structured questionnaires, physical examination, HIV-1/2 testing (Capillus, Trinity Biotech, Bray, Co Wicklow, Ireland), posterior-anterior chest X-rays, Ziehl-Neelsen staining of two independent sputum smears, and culturing of *M. tuberculosis* on Loewenstein-Jensen agar with subsequent determination of mycobacterial species, lineages and fine-typing of mycobacterial genotypes by spoligotyping, IS*6110* and determination of drug resistances fingerprinting as described previously [23]–[25]. Cases were HIV-negative and had lesions characteristic of pulmonary TB on the chest X-ray films. Patients were treated in the framework of the DOTS programme (Directly Observed Treatment Short-Course Strategy) organized by the National Tuberculosis Programme of Ghana.

Characterisation of controls included a medical history and clinical examination, chest X-ray and a tuberculin skin test (Tuberculin Test PPD Mérieux, bioMérieux, Nürtingen, Germany). 2217 individuals were PPD-positive and 129 individuals were PPD-negative. The group of PPD-negative controls comprised, to the large majority, household contacts of cases. The final group of PPD-negative controls consisted of 129 individuals. The controls had no radiological signs of actual or previous pulmonary TB. Further details of the recruitment procedure, the composition of the study group including the distribution of ethnicities, and application and interpretation of the PPD test have been described previously [25]–[28]. A large subset of the entire study group was genotyped for *IL10* promoter variants in the present study.

### Variants selected for genotyping

The selection of *IL10* promoter variants for genotyping was based on the functional relevance with regard to the extent of IL-10 production of the variant at position −2849 (rs6703630) and the proximal promoter haplotypes resulting from variability at positions −1082 (rs1800896), −819 (rs1800871) and −592 (rs1800872).

### Testing for population stratification

To assess whether population stratification was a major concern in our study, the median of the Armitage's trend test statistics of 61 unlinked biallelic markers (see Supplementary Table S1) was analysed to estimate the inflation factor λ for genomic control [29]. The inflation factor was calculated by dividing the observed median of the statistics trend tests by the predicted median of the χ^2^ statistics of 0.456 if inflation is absent.

### Genetic Analysis

After DNA extraction from peripheral blood by a magnetic separation technology (AGOWA® mag Maxi DNA Iisolation Kit, Berlin, Germany) according to the manufacturer's instructions, the *IL10* SNPs −2849, −1082, −819 and −592 variants were analysed by dynamic allele specific hybridization with fluorescence resonance energy transfer (FRET) in a LightTyper device (Roche Diagnostics, Mannheim, Germany). Primer pairs and sensor/anchor oligonucleotides for LightTyper-based *IL10* genotyping are listed in Table 2.

**Table 2:** Primer pairs and sensor/anchor oligonucleotides for LightTyper-based *IL10* genotyping.

*IL10* variant	rs number	primer oligonucleotides	sensor/anchor oligonucleotides
*IL10* −2849 A/G	rs6703630	F–TTAGCTACACATTTCAGAACAAATAAAGA	S–CCTCCCAGAGTGCTGAGATTACAGGC
		R–TTTTTTTTGTATTTTTATTAGAGAGGGGT	A–ATGATCCGCCCGCCTTG
*IL10* −1082 A/G	rs1800896	F–ATCCAAGACAACACTACTAAGGC	S–CCCTACTTCCCCCTCCCAAA
		R–GGGTGGGCTAAATATCCTCAA	A-GGATAGGAGGTCCCTTACTTTCCTCTTACC
*IL10* −819 C/T	rs1800871	F–ATCCAAGACAACACTACTAAGGC	S–AGGCACAGAGATATTACATCACCT
		R–GGGTGGGCTAAATATCCTCAA	A–ACAAGGGTACACCAGTGCTAACTGA
*IL10* −592 C/A	rs1800872	F–ATCCAAGACAACACTACTAAGGC	S–GCTTCCTACAGTACAGGC
		R–GGGTGGGCTAAATATCCTCAA	A–GGGTCACAGGATGTGTTCCAGGC

F, forward primer; R, reverse primer; S, sensor; A, anchor.

### IL-10 plasma levels

IL-10 plasma levels of patients homozygous for the *IL10* −2849/−1082/−819/−592 haplotypes GGCC and AACC were measured by the “Human IL-10 ELISA Ready-Set-Go” kit (Biocarta, Hamburg, Germany) according to the recommendations of the manufacturer. The range of detection was between 2 and 300 pg/ml.

### Databases and statistical analyses

Demographic data, self-reported signs and symptoms and primary laboratory results were double-entered into a Fourth Dimension database (San José, CA, USA). Microbiological data were provided as datasheets. All data were locked before using them in a pseudonymized form for further analyses.

Power calculation was performed with the CATS software (available at http://www.sph.umich.edu/csg/abecasis/CaTS/). The STATA 9 software (Stata Corporation, College Station, TX, USA) with supplementary modules (http://www-gene.cimr.cam.ac.uk/clayton/software/stata/genassoc) was used to calculate Hardy Weinberg equilibria (HWE) and odds ratios (OR) of IL-10 genotype frequencies. Logistic regression analyses (STATA 9) were applied to adjust for gender, age and ethnic groups. Haplotype frequencies and odds ratios (OR) with global and adjusted *P* values (10 000 permutations) were estimated and compared with the public “UNPHASED” software (version 3.0.12; http://www.mrc-bsu.cam.ac.uk/personal/frank/software/unphased/).The inflation factor for genomic control was calculated using the PLINK software (version 1.0.5; http://pngu.mgh.harvard.edu/~purcell/plink/download.shtml).

The nonparametric Mann-Whitney U test (STATA 9 software) was used to determine differences of IL-10 plasma levels in individuals homozygous for either the IL-10 −2849/−1082/−819 AAC (n = 25) or the GGC (n = 22) genotype.

## Results

### Power of the association study; Hardy-Weinberg equilibrium

A power of detection of >90% was achieved for both additive and multiplicative models, assuming an approximative TB prevalence of 0.004 in West Africa, a frequency of 0.1 for high risk alleles and a genotype relative risk of 1.4 (α = 0.001) with our sample size. The frequencies of the *IL10* −2849, −1082, −819 and −592 variants were tested and adjusted for gender, age and ethnicity in a large TB case-control sample from Ghana. Genotype frequencies did not deviate from Hardy-Weinberg equilibrium among cases and controls with the exception of *IL10* −2849 in cases (p = 0.02).

### Population stratification

The estimated inflation factor for genomic control of λ = 1.045, calculated by analysing 61 unlinked genetic markers, did not reveal a major population stratification in our study group.

### IL10 promoter genotypes

Statistical analyses were performed to compare the occurrence of *IL10* promoter genotypes between cases and all controls, cases and PPD-negative controls, cases and PPD-positive controls and between the combined group of cases plus PPD-positive controls and PPD-negative controls. Ethnicities did not influence any statistical analysis. After adjusting *P* values for gender, age and ethnicitiy and correction for multiple testing for the subgroups of the study population and the four variants tested, genotype frequencies did not differ significantly between groups (Table 3). A trend, however, was observed for the *IL10* −2849AA genotype, which occurred at higher frequencies among PPD-positive controls and in the combined groups of cases plus PPD-positive controls than in PPD-negative controls with an OR of 3.10 (CI 1.1–8.6, nominal *P* value 0.03), and an OR of 2.84 (CI 1.0–7.8, nominal *P* 0.04), respectively. No significant results were obtained when stratifying TB cases for whom genotyping results were available (n = 1587) for the three mycobacterial species *M. tuberculosis* (n = 1097; 69.1%), *M. africanum* (n = 480, 30.3%) and *M. bovis* (n = 10; 0.6%) that were identified after culturing of mycobacteria (data not shown).

**Table 3:** Genotype frequencies of *IL10* variants and odds ratios for comparisons of TB cases and controls, including stratification for PPD reactivity.

		cases	all controls	PPD− controls	PPD+ controls	cases plus PPD+ crtls	cases vs all controls		cases vs PPD− controls		cases vs PPD+ controls		PPD+ vs PPD− controls		cases plus PPD+ vs PPD− controls	
*IL10* variant	gt	%	%	%	%	%	OR (CI)	p	OR (CI)	p	OR (CI)	p	OR (CI)	p	OR (CI)	p
−2849	GG	50.2	52.4	60.2	52.0	51.1	1		1		1		1		1	
	AG	42.8	38.9	36.7	39.0	40.8	1.15 [1.0–1.3]	**0.028**	1.40 [1.0–2.0]	0.081	1.14 [1.0–1.3]	**0.047**	1.19 [0.8–1.7]	0.355	1.29 [0.9–1.9]	0.181
	AA	7.0	8.7	3.1	9.0	8.1	0.87 [0.7–1.1]	0.260	2.51 [0.9–7.0]	0.079	0.84 [0.7–1.1]	0.139	3.10 [1.1–8.6]	**0.030**	2.84 [1.0–7.8]	**0.044**
		n = 2001	n = 2330	n = 128	n = 2202	n = 4203										
−1082	AA	51.5	52.1	49.2	52.3	51.9	1		1		1		1		1	
	AG	40.9	39.8	39.8	39.8	40.3	1.03 [0.9–1.2]	0.684	0.97 [0.7–1.4]	0.886	1.03 [0.9–1.2]	0.645	0.92 [0.6–1.4]	0.690	0.94 [0.6–1.4]	0.752
	GG	7.6	7.1	11.0	7.9	7.8	0.92 [0.7–1.2]	0.545	0.67 [0.4–1.2]	0.202	0.95 [0.7–1.2]	0.715	0.69 [0.4–1.3]	0.241	0.68 [0.4–1.2]	0.205
		n = 1541	n = 1968	n = 128	n = 1840	n = 3381										
−819	CC	33.3	33.7	34.1	33.7	33.5	1		1		1		1		1	
	TC	49.4	47.8	49.6	47.6	48.4	1.04 [0.9–1.2]	0.646	1.05 [0.7–1.6]	0.825	1.04 [0.9–1.2]	0.642	1.00 [0.7–1.5]	0.982	1.03 [0.7–1.5]	0.886
	TT	17.3	18.5	16.3	18.7	18.1	0.94 [0.8–1.1]	0.552	1.15 [0.7–2.0]	0.619	0.93 [0.8–1.1]	0.464	1.26 [0.7–2.2]	0.406	1.21 [0.7–2.1]	0.473
		n = 1544	n = 1972	n = 129	n = 1843	n = 3387										
−592	CC	31.3	33.2	32.1	33.3	32.5	1		1		1		1		1	
	AC	51.9	48.2	50.0	48.0	49.7	1.18 [0.9–1.5]	0.145	1.17 [0.7–2.1]	0.586	1.19 [0.9–1.5]	0.137	0.90 [0.5–1.6]	0.694	1.00 [0.6–1.7]	0.993
	AA	16.8	18.6	17.9	18.7	17.8	1.04 [0.8–1.3]	0.776	1.01 [0.6–1.8]	0.978	1.03 [0.8–1.3]	0.791	0.98 [0.5–1.8]	0.940	0.98 [0.5–1.7]	0.942
		n = 1025	n = 1445	n = 106	n = 1339	n = 2364										

Nominal *P* values (p) and odds ratios (OR) adjusted for gender, age and ethnic groups by logistic regression. *P* values indicating a trend of association are highlighted. gt, genotype; PPD−, PPD-negative; PPD+, PPD-positive; CI, 95% confidence interval.

### IL10 promoter haplotypes

Reconstruction of haplotypes was performed with the UNPHASED software (Table 4). As reference haplotype for calculations of ORs and 95% confidence intervals (CI), the *IL10* haplotype −2849G/−1082G/−819C/−592C (GGCC) was chosen, as this haplotype is, most likely, associated with highest production of IL-10. For the comparisons that yielded significant global *P* values and that were stable after corrections for multiple testing (10 000 permutations calculated with ethnicity included as a covariate), haplotype-specific ORs were determined. The *IL10* haplotype −2849A/−1082A/−819C/−592C (AACC), which is most likely associated with low IL-10 production, was significantly less frequent in PPD-negative controls than in the group of cases (9.7% versus 15.5%; OR 2.15, CI [1.3–3.6]). A corresponding distribution of haplotype frequencies was observed when comparing PPD-negative controls with the combined groups of cases plus PPD-positive controls with (9.7% versus 15.4%; OR 2.12, CI [1.3–3.5]) and PPD-negative and PPD-positive controls (9.7% versus 15.3%; OR 2.09, CI [1.2–3.5]). The distribution of haplotypes in individuals infected with either *M. tuberculosis*, *M. africanum* or *M. bovis* did not differ when compared to controls and subgroups of controls.

**Table 4:** Frequencies of *IL10* −2849/−1082/−819 promoter haplotypes and comparisons between subgroups.

*IL10* haplotype			cases	all controls	PPD+ controls	PPD− controls	cases plus PPD+ controls	cases vs all controls	cases vs PPD+ controls	cases vs PPD− controls	PPD+ vs PPD− controls	cases plus PPD+ vs PPD− controls
−2849/−1082/−819			%	%	%	%	%	OR (CI)	OR (CI)	OR (CI)	OR (CI)	OR (CI)
G	G	C	14.1	14.6	14.3	18.9	14.2	1	1	1	1	1
G	A	C	14.6	14.8	14.5	19.6	14.5	1.02 [0.8–1.2]	1.02 [0.8–1.2]	1.00 [0.6–1.6]	0.98 [0.6–1.6]	0.99 [0.6–1.6]
G	A	T	41.7	42.2	42.4	40.0	42.0	1.02 [0.9–1.2]	1.00 [0.9–1.2]	1.40 [1.0–2.0]	1.40 [1.0–2.0]	1.40 [1.0–2.0]
A	G	C	14.2	13.5	13.6	11.9	13.9	1.09 [0.9–1.3]	1.06 [0.9–1.3]	1.59 [0.9–2.7]	1.51 [0.9–2.6]	1.55 [0.9–2.6]
A	A	C	15.5	14.9	15.3	9.7	15.4	1.08 [0.9–1.3]	1.03 [0.9–1.2]	2.15 [1.3–3.6]	2.09 [1.2–3.5]	2.12 [1.3–3.5]
			n = 2968	n = 3866	n = 3622	n = 244	n = 6590	p_global_ 0.85	p_global_ 0.96	p_global_ **0.009**	p_global_ **0.012**	p_global_ **0.009**
										p_global/corr_ **0.013**	p_global/corr_ **0.017**	p_global/corr_ **0.012**

Only haplotypes with frequencies >0.01 are given. The reference haplotype GGC is associated with the highest IL-10 production with the following haplotypes ordered according to their presumed decreasing IL-10 production [25]. The variant occurring at position −592 may in all cases be inferred from those at postions −1082 and −819.

n, estimated counts of inferred haplotypes in cases and controls; PPD+, PPD-positive, PPD−, PPD-negative; OR, estimated odds ratios of a haplotype, compared to the reference haplotype; CI, 95% confidence interval. pglobal refers to the overall likelihood ratio test of association; pglobal/corr represents adjusted P values after 10 000 permutations.

As estimated haplotype frequencies should not be treated as observed data and in order to further determine the empirical significance of our findings, the global *P* value of differences in haplotype frequencies was verified by calculating 10 000 permutations. The adjusted and corrected global *P* values p_global/corr_ remained significant for comparisons of PPD-negative controls with cases (p_global/corr_ = 0.013), PPD-negative controls with the combined group of cases plus PPD-positive controls (p_global/corr_ = 0.012) and PPD-negative controls with PPD-positive controls (p_global/corr_ = 0.017). The associations held true when including ethnicity as a covariate.

### IL10 plasma levels

**Figure 1:**
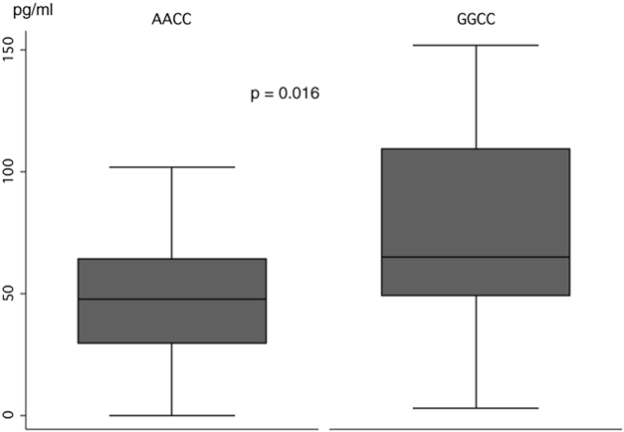
IL-10 plasma levels (pg/ml) in individuals homozygous for the presumed “low” and “high producer” haplotypes −2849A/−1082A/−819C (AAC) and −2849G/−1082G/−819C (GGC). Concentrations differ significantly between AAC and GGC carriers (p = 0.016; Mann-Whitney U test).

In order to confirm that the combination of −2849G and the haplotype −1082G/−819C/−592C is in fact associated with higher Il-10 plasma levels, concentrations of IL-10 were determined in patients homozygous for either GGCC (n = 22) or AACC (n = 25) by an enzyme linked immunosorbent assay before the initiation of antimycobacterial treatment. Significantly higher plasma levels of IL-10 (p = 0.016; Figure 1) could be confirmed to occur among carriers of GGCC compared to AACC. Mean plasma levels of GGCC and AACC cariers were 74.4 pg/ml (CI [56.6–92.2]) and 46.9 pg/ml (CI [35.6–58.1]), respectively.

## Discussion

The numerous studies undertaken to identify genetic correlates of resistance and susceptibility to pulmonary TB have provided inconsistent evidence of a few candidate genes that exert minor influences only. In order to ascertain true exposure to *M. tuberculosis*, most of the genetic studies conducted so far have focused on comparisons of cases with PPD-positive controls. Although a substantial body of immunological *in vitro* findings suggests that innate resistance of humans to *M. tuberculosis* may occur, it has received by far less attention in genetic association studies.

Studies of the proximal *IL10* −1082G/A, −819C/T, −592C/A promoter haplotypes in different populations have shown that, at these positions, only the haplotypes GCC, ACC and ATA occur. The few studies reporting on other than these haplotypes in China and Thailand (ATC, ACA) [30] await confirmation. Genotyping of the variants at positions −1082 and −819 allows, therefore, to include in haplotype reconstruction the variant at position −592 [14]. In order to ascertain the preferential occurrence of these haplotypes in our African study population, we have genotyped the *IL10* −592 nucleotide in a subsample of our study group (n = 2470) and confirmed those haplotypes with the UNPHASED haplotype estimation algorithm.

It is also established that the three haploytypes differentially affect the transcriptional activity of the *IL10* gene. High, intermediate and low IL-10 production is associated with the proximal *IL10* promoter combinations GCC, ACC and ATA, respectively [14], [16]. Compared to the *IL10* −2849GG genotype, the AA genotype is strongly associated with lower IL-10 production, as is the proximal ATA haplotype [31], [32]. The relevance for IL-10 production of the *IL10* −2849 variants has, to our knowledge, not been assessed together with that of the proximal *IL10* −1082/−819/−592 haplotypes. Based on the functional results available so far for the distal *IL10* −2849 variant and the proximal haplotypes it was, however, conceivable that the *IL10* −2849A/−1082A/−819C/−592C (AACC) haplotype that we observed at higher frequencies among cases and PPD-positive controls than in PPD-negative controls causes rather low constitutional IL-10 levels. Significantly higher IL-10 plasma levels of homozygous carriers of the *IL10* GGCC haplotype, compared to homozygous AACC carriers, were confirmed by the determination of IL-10 plasma levels in the group of TB patients (Figure 1). The IL-10 concentrations in plasma that were measured before the initiation of specific antimycobacterial treatment were similar to those reported previously [33], [34]. The haplotype most likely associated with lowest IL-10 production, AATA, was observed at lowest frequencies only in our study population that did not allow further analyses.

The frequencies of the *IL10* −2849 genotypes were in HWE among controls, but not among cases. Notably, the frequencies were in a sample from Ghana and in African-Americans almost identical to those observed in our study [35], [36] and they were largely similar in a population of European descent [30]. Associations with the *IL10* −2849 polymorphism or with haplotypes comprising this variant have also been described in other conditions, e.g. in rheumatoid arthritis, decreased female fertility, systemic sclerosis, pre-eclampsia, and in leprosy [37]–[41].

Our findings indicate that individuals exists who are exposed to *M. tuberculosis* infection, but are genetically distinct from both PPD positives and TB cases. This observation substantiates epidemiological evidence that even heavy exposure to *M. tuberculosis* of healthy individuals in closed environments may result in a lack of tuberculin skin response [5], [6]. We have, in our study, included PPD-negative controls who were exposed to *M. tuberculosis* transmission and have observed the greatest difference of haplotype frequencies in the comparisons of PPD-negative controls with TB cases and PPD-positive controls. The haplotype that is, based on the aforementioned functional studies, most likely associated with low IL-10 production (−2849A/−1082A/−819C/−592C; AACC) was less frequent among PPD-negative controls than among cases and PPD-positive controls, including the combined groups of cases plus PPD-positive controls, compared to the reference haplotype GGCC which is associated with high IL-10 production. Notably, and with respect to the presumed decreasing extent of IL-10 production of the different haplotypic combinations given in Table 3, ORs of comparisons of haplotype frequencies increased gradually from the high-producer GGCC haplotype to the low-producer haplotype AACC in those comparisons that provided significant global and corrected *P* values (cases vs. PPD-negative controls, PPD-positive vs. PPD-negative controls, cases plus PPD-positive vs. PPD-negative controls; Table 3).

The pro-inflammatory response that is initiated by *M. tuberculosis* is antagonized by anti-inflammatory mechanisms. After phagocytosis of the pathogen, macrophages, T-lymphocytes and other cells produce IL-10 [42]–[44]. Among further determinants involved, the extent of IL-10 production depends largely on the genetic composition of the promoter haplotype. The overall consequences of IL-10-dependent immunological mechanisms are typically dose-dependent. In an early phase of the infection, IL-10 attenuates mechanisms that cause activation of adaptive immune effector cells [45]. At the same time, antigen presentation is impaired by retarded maturation of antigen presenting cells that are infected with mycobacteria. Inflammation is confined and the generation of adaptive immunity is postponed [46]. The effects exerted by IL-10 may result in antigen-specific anergy [47], which is compatible with PPD negativity in the present study. Such a state of a delayed and decelerated development of specific adaptive responses enhances local effects of innate immune mechanisms that, to a certain degree, may successfully control a pathogen. When initial clearance of pathogens by innate immunity fails, inflammation progresses and T-cell responses develop differentially, resulting in either PPD positivity or in manifest pathology.

We believe that the lower frequency of the *IL10* promoter haplotype AACC among PPD-negative individuals reflects functional differences between PPD-positive and PPD-negative control individuals rather than between cases and PPD-negative controls. PPD-negativity might result from IL-10-dependent T-cell suppression, and the actual levels of IL-10 are likely to determine either T-cell reactivity or anergy. Antigen-specific tuberculin anergy has been shown to occur in patients during active TB and persisting even after successful treatment [1], [4], and higher IL-10 levels have been documented in these patients when compared to PPD-positive individuals. Since the cases of our study did not undergo tuberculin testing, we cannot exclude IL-10-dependent PPD negativity in a subgroup of our cases. It is conceivable that the rate of PPD negativity in our case-group corresponds to that of the total control-group and that haplotype-associated dose-dependent IL-10 effects in cases and controls may cause either PPD-negativity or PPD-positivity.

Since the association that we observed does not apply to the entire study group but only to a subfraction as tuberculin tests were not performed on cases, the *IL10* promoter haplotypes GGCC and AACC are certainly not surrogate markers for the assessment of disease risk or the quality and extent of the skin test response after exposure to *M. tuberculosis* antigens. However, and with regard to the sample size and the robustness of case and control phenotypes, in particular the exposure of PPD-negative controls to *M. tuberculosis*, our results make a contribution of *IL10* promoter haplotypes to the polygenic mosaic of TB susceptibility likely. An adjusted equilibrium of pro- and anti-inflammatory mechanisms exerted by IL-10 appears to depend at least in part on the individual genetic profile of its promoter.

## Supporting Information

Table S1 (0.03 MB XLS)
